# Forever young: China’s migration regime and age patterns

**DOI:** 10.1080/15387216.2023.2279545

**Published:** 2023-11-12

**Authors:** Xiaxia Yang, Kam Wing Chan

**Affiliations:** a Lau China Institute, King’s College London, London, UK;; b Department of Geography, University of Washington, Seattle, WA, USA

**Keywords:** Age, migrant settlement, temporary migration, *hukou* system, China, migrant flow and stock

## Abstract

Chinese institutional arrangements, particularly the *hukou* system, hinder long-term settlement of internal migrants by limiting their access to social benefits. This article proposes a new method for assessing migrant settlement: the use of age data to investigate the link between migrant “flow” and “stock”. We contend that migrants’ inability to settle mainly derives from two sources: the difficulties in maintaining migrant family togetherness, and the impediments to long-term residence of migrants themselves. Age-related indices were developed to compare China’s internal migration with other countries’ internal and international migration. The results indicate a “China difference” in migration age patterns – child and elderly dependents of migrant workers are discouraged from migrating, while migrants growing old tend to return to the origins than to remain in the destinations. Consequently, family togetherness and long-term residence in the destinations are often unachievable for migrants. Our analyses highlight China’s unique migrant labor regime, where temporary migrant workers are continuously “recycled” to keep destinations’ workforce “forever young”, reducing production costs of Chinese goods in global markets. Methodologically, our age-based “mobile-to-settled” transition framework and “settlement rate” of migrants in the transition are of value in examining migrant settlement chances more generally, applicable to internal and international migration beyond China.

## Introduction

China’s massive internal migration has been widely associated with its rapid economic rise in the past four decades, especially as it has become the “world’s factory” (Lee 1998). Among the attributes making China’s economic engine tick is the youthfulness of its migrant labor, which has been noted before (e.g. Chan 2010; Hu 2021) but not systematically investigated. To illustrate, for China, the percentage of population aged 15–59 in 2020 was 63.4% (NBS 2022). Whereas for Shenzhen and Guangzhou, two of China’s major manufacturing hubs, that figure was 79.6% and 74.7%, respectively (GBS 2022), 11–16% higher than the national average. As will be examined below, the core industrial cities in China absorb massive migrant labor, which is overwhelmingly dominated by young adults. The feat of maintaining such a youthfulness underscores the power of these premier “cities of migrants”, notwithstanding the general and rapid aging of China’s total population (Davis 2014). More importantly, these cities are able to continuously “recycle” young migrants so that their migrant labor force never “grows old” over time, reaping a huge “demographic dividend” of age (Cai 2016).

This paper examines the unique feature of “forever youthfulness” of China’s internal migrants. We use internal and international migration of other countries as reference points to generate insights into China’s special migration age patterns. Specifically, we look at three countries, India, Japan, and the United States (US), to compare their internal migrants’ age patterns with China’s. All three have comparable age data of internal migrants available to serve our research needs and, despite differences in their demographic features, share commonalities that allow for meaningful comparisons, as will be shown later. Moreover, several scholars have pointed out that institutional barriers inside China resemble international border controls (Bao et al. 2011; Johnson 2017; Roberts 1997). Given this, we expect the age patterns of China’s internal migration to share commonalities with that of international immigration, and we draw on a comparison with international immigration to the US to explore this. The US has a huge number of immigrants, and the age data for them is accessible. By comparing with these reference points, we highlight the similarities and deviations of China’s migration from others. In particular, we think age data open a new window to study the barriers Chinese migrants face in settling in the destinations – migrants’ being “forever young” suggests they are not able to stay for long periods of time and grow old in the destinations. Based on the demographic concepts of migrant “flow” and “stock”, we develop methods and indicators that use publicly obtainable age data to measure migrant settlement. Compared to sample surveys often used to study migrant settlement patterns, age data are both more available and more extensive in coverage. Furthermore, studying the (in)capacity of migrants to settle pinpoints the direct and intimate relationship between China’s development model in the reform period and its internal migrant labor system.

The paper is structured as follows: In the next section, we put forward two research questions that relate migrants’ “youthfulness” to their (in)ability to settle in the destinations. One question is on family togetherness, and the other is on long-term residence. We posit that these two vital components of settlement are impeded for many migrants in China. In reviewing the relevant literature, we examine the transient nature of much of China’s migration, the institutional basis of this nature especially the *hukou* system, and its echoes of China’s development strategy. We then explain our research method that taps into age data of the “flow” and “stock” to study migrant settlement. We explore the mechanism of the “mobile-to-settled” transition and associate it with the age profiles of migrant flow and stock. Among the various indicators we develop is the migrants’ “settlement rate”, which is the proportion of migrants who make the “mobile-to-settled” transition, as a way to quantify the settlement opportunity of migrants. The methodology section is followed by empirical data analyses. We decompose the two research questions into four expectations. To test these expectations and foreground the uniqueness of China’s migration system, we contrast the age patterns of Chinese migrants with that of internal and international migrants in other countries.

## Research questions: age and the temporariness of China’s internal migration

**Figure 1.**
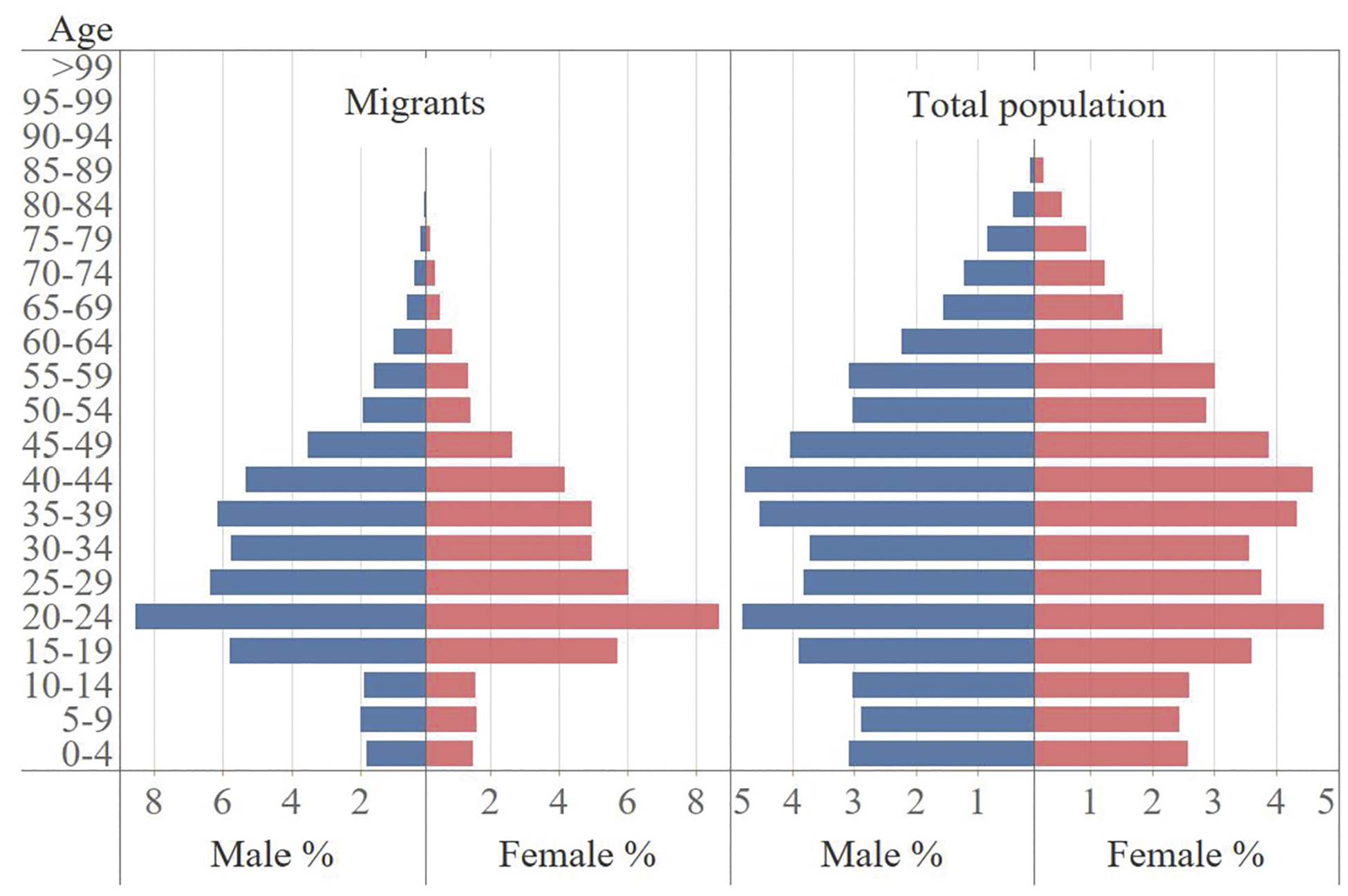
Age pyramids of migrants and total population in China, 2010. Source: National Bureau of Statistics, China. *2010 Decennial Census*.

The internal migration of China in the reform era has been widely considered the largest in human history (Liang 2016; Miller 2012). “Migration” is large in China, but it is also special. The great majority of “migrants” in China in the reform era only stay temporarily in the destinations; they are more like circulators than true migrants (Chen and Fan 2018; Fan, 2008; Roberts 1997). A vital contributor to this phenomenon is the *hukou* (household registration 户口) system, an institution that functions as a migration control and social benefits distribution system (Chan 2009; Whyte 2010). One’s *hukou* is attached to a place, and only local *hukou* holders have full access to the key public services, including education, health care, social housing, etc. Among them, access to education is especially crucial to migrant families. Many children of migrants were forced to be left behind in their *hukou* origins because attending public primary and secondary schools are difficult in the destinations, and they are almost barred from attending high school and taking college entrance exams there (Chan and Ren 2018; Gao 2014; Ling 2019). Consequently, unlike in other countries, there are two types of internal migrants in China, the *hukou* and non-*hukou* migrants. The *hukou* migrants are those who successfully register their *hukou* in the destinations and have full access to local benefits, while the non-*hukou* migrants are those who cannot do so. The bulk of migrants in the reform era belong to the latter type (Cai, Guo and Wang 2016). Legally considered temporary, their movement is called “floating” (*liudong* 流动) – they are classified by the National Bureau of Statistics of China (NBS) as “floating population” (*liudong renkou* 流动人口) to indicate their temporariness. Chinese migrants’ being “floating” differs from international undocumented migrants’ being “illegal” in that the non-*hukou* migrants can move freely without being detained or deported. Detention and repatriation were common for migrants in China without proper documentation before 2003, yet the death of a young migrant named Sun Zhigang prompted a shift of the policy. On the other hand, the two are also quite similar, in that the legal boundaries of the destinations (provincial or city boundaries in the former case, and national boundaries in the latter case) dictate migrants’ access to a myriad of benefits and thus their citizenship (Johnson 2017). Statistically, according to the NBS, the floating population are those who have left their *hukou* origins for at least six months. In this article, we refer to them as “migrants”, as they are generally in China, and they are the focus of our analyses. As shown in Figure 1, their age pyramid is shaped like a “Christmas tree”, with young adults taking the largest share and a very narrow base and top, quite different from the total population of China.

This study aims at using age as a lens to investigate the legally enforced temporariness of China’s internal migration. We argue that age data attest to the fact that, with the lack of access to resources for the social reproduction of migrant labor, to the great majority of internal migrants in China – those without local *hukou* – migration is often temporary and does not lead to settlement. “Settlement”, of course, involves a range of issues. Nevertheless, “migrant settlement”, especially the constituent elements of “settlement” *per se*, is not a well-recognized area of research. One extensive body of literature closely related to this topic was the “integration” or “assimilation” of international migrants and their offspring into the host country (Maxwell 2010; Waters and Jiménez 2005). Migrants in this research context are usually from cultural, linguistic, or religious backgrounds different from the receiving society, and it takes time and effort for them to adapt to the new environment (Iredale, Hawksley, and Castles 2003). Another relevant strand of academic work evaluated the decision-making processes of migrants and their families from departure to settlement (Erdal and Ezzati 2015; Friberg 2012). A considerable amount of research on China’s internal migration fell in this category, and they looked at the “settlement intention” of migrants (e.g. Liu and Wang 2020; Yang and Guo 2018; Zhu 2007). Among the few scholars interested in the substantive aspects that comprise the “settlement” of migrants in the destinations, which is of our concern, were Seol and Skrentny (2009). In their research that investigated the rare international migrant settlement in East Asia, they identified “family reunification” as a vital dimension of settlement. Additionally, according to Massey’s (1986) analysis of the settlement process among Mexican migrants to the United States, spending time in the destinations, either through continuous residence or repeated trips, is the precondition of migrants’ transition from “sojourners” to “settlers”. Combining these claims and the general observations on China’s internal migration, we posit that the barriers to settlement in China usually come from two sources: the difficulties in maintaining migrant family togetherness, and the impediments to long-term residence of migrants themselves. Two research questions are formulated reflecting the two sources of settlement barriers:

1. Can family togetherness be maintained during migration in China? We expect that only working-age adults are able to migrate to work, while dependent population, including children and the elderly, are discouraged from migrating.
2. Can migrants stay in the destinations for a long time in China? We expect that as (initially young) migrants age over time, they tend to return to the origins instead of remaining in the destinations.

In answering these questions, we show how migrant settlement can demographically manifest itself in age. The significance of doing so lies in that migrant settlement cannot be easily quantified on a large scale. Previous attempts to empirically assess migrant settlement largely depend on sampling and statistical inference but not a direct measurement of the general population (e.g. Fan, 2011; Massey 1986), as the latter obviously involves too much work. The use of age data largely resolves the issues of data availability and coverage, which is thus an effective shorthand means of examining migrant settlement.

Our two research questions point to the rights to reside and bring family members, both of which are key to settlement. Furthermore, they point to China’s “low-cost” (mainly in the short term) development strategy closely connected to its migration regime. Through denial of access to local public services, migrants are made producers of economic value, but not consumers of destination social services. Child and elderly dependents of migrant workers, as well as earlier migrants who grow old, are the ones who need social services the most. By excluding them, the major destinations, usually the coastal industrial and trade centers, significantly lower their public spending (Zhang and Li 2016). This development strategy saves the social reproduction costs of migrant labor for both the local governments and employers at the destinations, thus reducing the production costs of Chinese goods and ensuring their unrivaled competitiveness – the “China price” – in global markets (Harney 2008). Destinations constantly replenish their pool of cheap labor with working-age adults, who only stay for their youthful years for work but not to raise a family or retire. In this way, they have enjoyed a long period of almost indefinite supply of “golden mine” of young migrant labor reserve, or the “demographic dividend”, until, of course, when overall population aging kicks in (Chan 2010). In other words, China’s migrant labor regime operates through continuous “recycling” of young migrant workers, thereby keeping the destinations’ workforce “forever young” and the direct and indirect labor costs low. Of course, beyond the lack of access to urban welfare, other factors also contribute to the reluctance of migrants to bring their child and elderly dependents and get settled. Many migrants find it difficult to afford the cost of raising a family in the urban destinations with low wages; nor do they have time to care for their children with long working hours. However, the difficulties of enrolling their children in schools in the destinations are the major consideration for most (Chan and Ren 2018; Ling 2019). Many migrants are also reluctant to give up their rural *hukou* and hence their rights to rural land in exchange for *hukou* in smaller cities, where the social benefits are limited (Gu et al. 2020). They would love to settle in big cities, but the *hukou* door of those cities are totally shut off to them (Chan 2023). In short, while some of the factors that prevent migrants’ settlement in Chinese large cities may look similar to those found elsewhere in other countries on the surface, as pointed out by Chan and Wei (2019), the primary driver of the various difficulties faced by migrants is closely related to their lack of local *hukou* and the resultant second-class citizenship status.

While arriving migrants are typically young everywhere, in most countries where migration is “free” – which specifically refers to the absence of a *hukou* equivalent in this paper – most manage to get settled eventually in the destinations. They gradually become “locals” over time, raising a family or bringing one from the origins, as recounted in the *Arrival City* stories by Saunder (2012). Corresponding to the above two research questions, we expect that in those “free” systems, family togetherness and long-term residence of the migrants can be better achieved. Dependents of current migrants, including children and the elderly, as well as earlier migrants who grow old, will not be discouraged from staying in the destinations. From this, we can further explore if China’s migration system is distinctive from the others. In short, comparisons with other countries will shed light on the “China difference” of migrant settlement and labor regime.

## Conceptual design and methodology: linking migration to settlement by examining age data of migrant flow and stock

This section explains our conceptual design that relates age to migration and settlement. We begin by showing what the age profiles of migrant “flow” and “stock” generally would look like under “free” migration, followed by the “mobile-to-settlement” transition from the flow to the stock, and how we numerically measure the settlement chance of migrants through this transition.

### Age profiles of migrant flow and stock

In demography, migration can be measured in two ways, the “flow” and the “stock”. The “flow” refers to the migrants entering or leaving a place during a certain fixed time period. The “stock”, on the other hand, is a snapshot of all the migrants in the destination at a certain time point (Bartram, Poros, and Monforte 2014). The stock is thus the accumulated total of net flows and attrition (deaths of migrants) of all the time in the past. Existing demographic literature on internal migrants’ age patterns almost exclusively covers the flow but not the stock (e.g. Raymer and Willekens 2008; Rogers 1978). There is a good reason for this: most countries do not have institutions like the *hukou* system that differentiate locals from domestic migrants and operate at a national, societal level. Instead, many of their internal migrants are initially “outsiders”, but over time (in some cases as soon as a few months), they gain rights and benefits similar to those of locals. Therefore, the censuses of most countries only report on the flow to show the change in population (by comparing previous and current addresses, for example) but not on the stock. An exception is where there is a relevant policy concern, such as in India, where internal migrant assimilation is a major public policy concern (Rajan and Bhagat 2021), and the Indian censuses have information on the stock. Simply put, data on internal migrant stock are nonexistent in most countries. In contrast, in China, non-*hukou* migrants always carry a legal “outsider” status once they leave their *hukou* registration place, which thus is a stock concept. While internal migrant stock data are commonly not collected, countries where international immigration is significant, such as the US, do collect immigrant stock data based on the “foreign-born” criterion.

**Figure 2.**
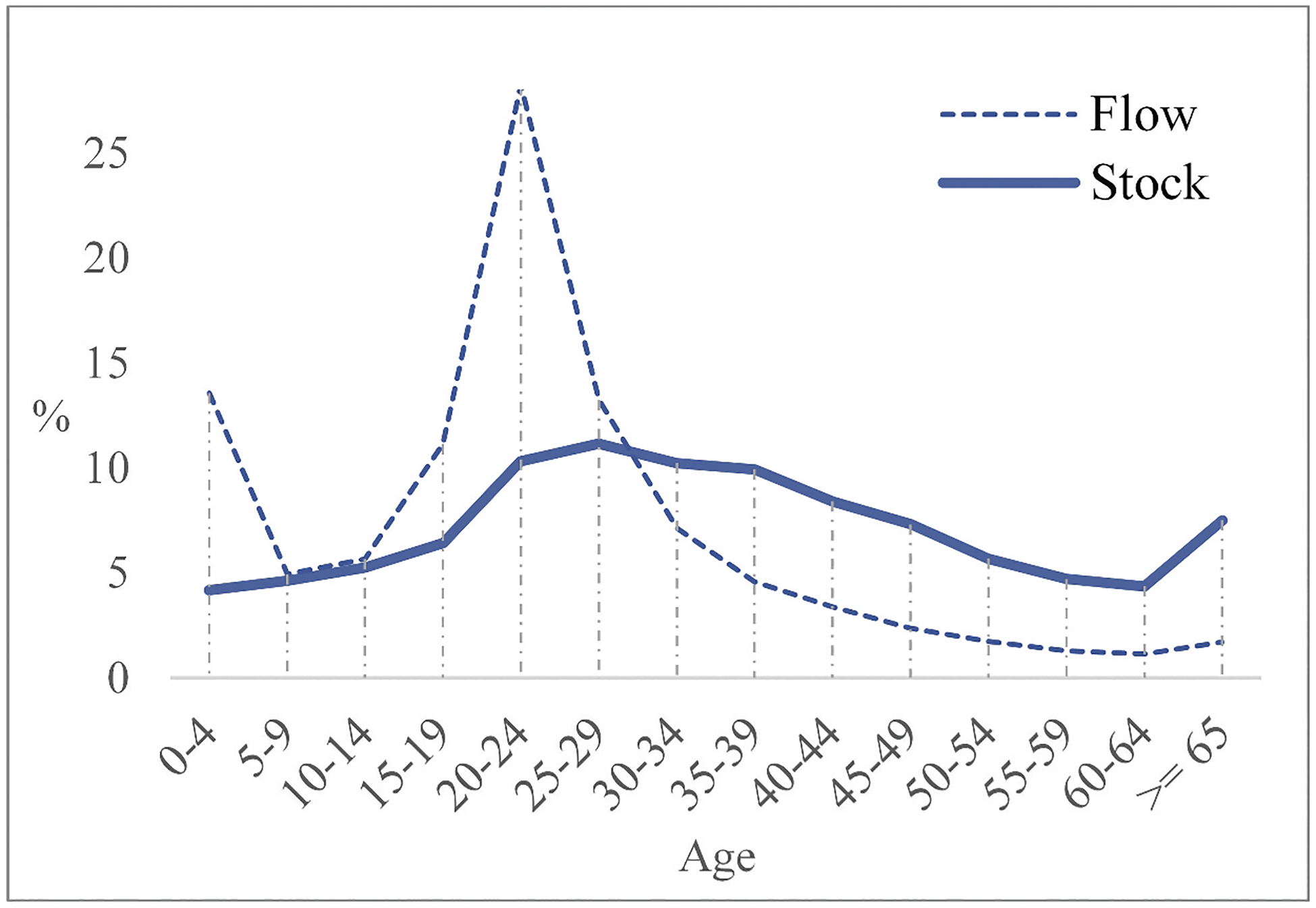
Age profiles of internal migrant flow and stock of India, 2011. Source: See Table 2

To set up a “paradigm” of internal migrants’ age profiles under “free” migration, against which China’s case can be compared, we draw on the case of India. As mentioned above, India has attempted to formulate policies regarding the inclusion of internal migrants. It is one of the few large countries whose census covers migrant residence duration, which enables the extraction of both flow and stock data. The age profiles of migrant flow and stock of India in 2011 (Figure 2) are presented by normalizing the age structures into percentages, the same for all age profiles throughout this paper. In the literature, migration age patterns were most commonly demonstrated by age-specific migration rates, i.e. the number of migrants divided by the total population of a specific age group (e.g. Menashe-Oren and Bocquier 2021; Rogers 1978). However, this study uses age structures of migrants to examine migration age regularities. The reason is that migration rates measure the propensity to move – they look at population who move within a time window, not the accumulation of population who have experienced migration anytime in the past. This suggests that migration rates can only apply to the flow but not the stock, which is a vital limitation for this study. On the other hand, the main disadvantage of age structures compared to age-specific rates is that the former cannot eliminate the influence of the age distribution of the total population, including both migrants and non-migrants. We account for this by incorporating age patterns of the selected countries’ total population into the analyses (more details in the Data Analysis section). Additionally, we normalize the age structures into percentages to remove the influence of different population sizes of the selected countries.

In the literature, a widely accepted “standard” age schedule of the flow has two peaks, a higher peak at young adult age and a lower peak at young child age (Raymer and Willekens 2008; Rogers and Castro 1981). The peak during young adulthood is caused by major life course events such as job changes, marriages, and giving birth. The peak in childhood is a derivative of parental migration, since young children tend to accompany their migrant parents. Consistent with the established knowledge, the higher peak at young adult age and the lower peak at young child age are evident on the flow curve in Figure 2, suggesting strong age selectivity of the flow. Yet the stock curve in Figure 2 is much flatter, suggesting only mild age selectivity of the stock. Since the stock is the accumulated total of all previous net flows and deaths, it involves the accumulation of different migrant flow cohorts and the aging effect of earlier cohorts over time. The different age selectivity of the flow and the stock thus indicates that surviving flows (i.e. net flows minus deaths) of multiple cohorts at different times, while highly age-selective at the point of migration, eventually fuse to form a stock of a much more evenly, smoothly distributed age pattern after migration has taken place for a long time.

### The “mobile-to-settled” transition examined by flow and stock age profiles

**Figure 3.**
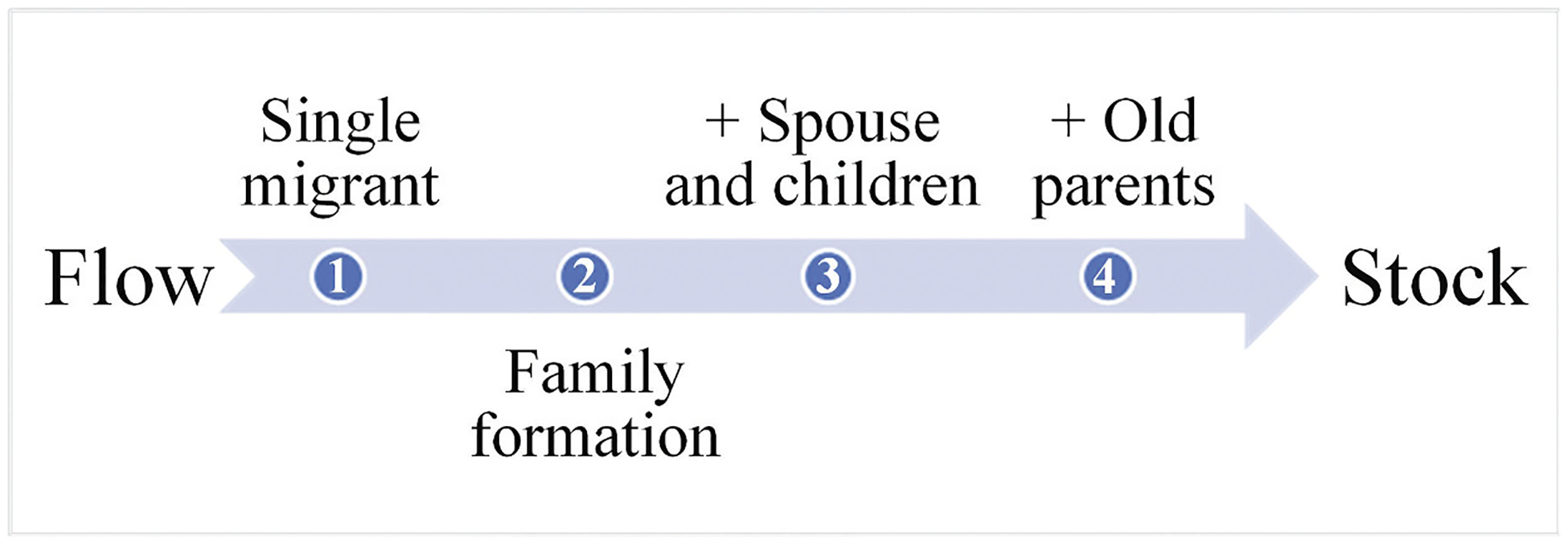
Life-course settlement process from migrant flow to stock in the “mobile-to-settled” transition.

Having interrogated the general age profiles of the flow and the stock under “free” migration, we turn to how they can be used to analyze the link between migration and settlement. As mentioned earlier, we contend that the two main sources of barriers to settlement in China are the difficulties in maintaining migrant family togetherness, and the impediments to long-term residence of migrants themselves. When these barriers are weak, migrants can stay in the destinations for a long time. Earlier cohorts in the migrant population will generally have experienced the formation and reunification of families. Figure 3 schematically illustrates the typical stages of settlement for a single flow (i.e. one migrant cohort) from a life-course perspective (Bernard, Bell, and Charles-Edwards 2014a). Such a life-course settlement process denotes a “mobile-to-settled” transition, with the flow indicating people’s movement and the stock indicating their stabilization. A pioneer migrant is typically a single young adult (stage 1 in Figure 3). Later, they get married and form a family (stage 2). If not impeded by institutional barriers, married pioneer migrants are joined first by their children (stage 3), and subsequently (or at the same time) by their old parents (stage 4). Overall, the age selectivity of pioneer migrants is much stronger than that of follower migrants, whose age range is much wider. This is because pioneer migrants are usually employment-oriented, yet follower migrants could be triggered by family reunion (Lindstrom and Ramírez 2010). In line with the life-course settlement process, in Figure 3, young adults are the main force of pioneer migration, showing up as the higher peak on the flow curve. Also, young children tend to be brought by their parents, manifested as the lower peak on the flow curve. As migrants from early cohorts grow old and reunite with other family members, the young-adult- and young-child-dominance in the age profile of the flow gradually tapers off and is replaced by a relatively evenly age distribution of the stock. Long-term family reunion along with population aging mitigates the short-term strong age selectivity, which explains why the stock curve appears considerably flatter and smoother than the flow curve.

In contrast to the above, China has strong institutional barriers to discourage settlement, which may impede migrant family togetherness and long-term residence. The chance of family reunion between migrants and their children and old parents (stages 3 and 4 in Figure 3) may be severely lowered. Given the limited access to education in the destinations, even children who are brought initially can be sent back to the origins (Ling 2019). Consequently, the young children’s peak in the flow curve could be quite small or even completely missing. Moreover, earlier migrants may not settle down over time; many would have left the destinations before growing old. As a result, the level of age selectivity among migrants could remain strong over time, so that the age selectivity of the stock continues to be strong, and the stock curve is not much flatter relative to the flow curve. In other words, migrants in China may hardly make the “mobile-to-settled” transition as those under “free” migration; many of them would have left the destinations before being counted in the stock.

### Settlement chance of migrants in the “mobile-to-settled” transition

To capture migrants’ chance of making the “mobile-to-settled” transition, i.e. their settlement chance, we develop an average, overall “settlement rate” index, *S*, whose calculation is based on available flow and stock data. *S* denotes the proportion of the flow population who remain in the destinations after a long time and become part of the stock population. Put another way, it is the “retention” rate of a migrant group (in the case of this study, a migrant group refers to all migrants in a selected country), using its current schedule of age-specific “staying” rate. We make some assumptions to simplify the calculations of *S* without scarifying its utility. First, we keep the age-specific fertility, mortality and net migration rates constant over time so that each migrant group has a constant age composition. In addition, we make *S* a single indicator that measures the “average” retention rate of a migrant group, keeping it the same across different age groups and over time. Given a known flow age distribution at the start and a known stock age distribution at the end, one can estimate *S* through a series of iterations.

To estimate *S*, we first define a “hypothetical” stock. With the actual flow being the base population and a known *S*, a stock after the “mobile-to-settled” transition can be computed. Such a stock is a “hypothetical” one because it is produced artificially. Put another way, if a reasonable *S* is estimated, the hypothetical stock should approximate the actual stock. One can simply try out a series of *S* values ranging from 0.1 to 0.9 and see which number graphically brings the hypothetical and actual stock curves together, as a broad estimation of *S*. To explain the specific computations of *S*, we start from the scenario when there is only one flow cohort. For age group *i* who remains in the destination after time *t*, the “settlement rate” *S* is:

(1) $$S=\frac{l_{i}}{l_{i-t}}$$

where *l*_*i−t*_ denotes the population aged (*i − t*) at time 0, and *l*_*i*_ denotes those aged *i* at time *t*.

Hence

(2) $$l_{i}=l_{i-t}S$$

The second scenario is when two flow cohorts arrive successively at time 0 and time *t*. Since we have assumed the age composition of the population to be the same over time, the aggregated population _*t*_*K*_*x*_ aged *x* at time *t* is:

(3) Ktx=lx+lx−tS

where *l*_*x−t*_ denotes the population aged (*x − t*) at time 0, and *l*_*x*_ denotes those aged *x* at time *t*.

Even if the transition from migrant flow to stock takes “infinite” time in theory, we find that after 5*t* time of change, the estimated *S* stabilizes at a certain number (when rounded to one decimal place); in other words, longer time periods, such as 6*t*, do not make much difference in the estimation of *S* in practice for our selected countries. Hence, we can reasonably consider that multiple flow cohorts amount to the hypothetical stock after 5*t*. Following equation (3), the aggregated population _5*t*_*K*_*x*_ aged *x* at time 5*t* would be:

(4) K5tx=lx+(lx−t+lx−2tSt+lx−3tS2t+lx−4tS3t+lx−5tS4t)St

which can be simplified into:

(5) K5tx=lx+lx−tSt+lx−2tS2t+lx−3tS3t+lx−4tS4t+lx−5tS5t

where *l*_*x*_*, l*_*x−t*_, ···, *l*_*0*_ are the population aged “65 and over”, “60–64”, ∙∙∙, “0–4” (this age grouping may vary according to the dataset) at time 0, which take the values of age composition of the actual flow. _5*t*_*K*_*x*_, _5*t*_*K*_*x−t*_, ···, *5tK*_*0*_ are the aggregated population aged “65 and over”, “60–64”, ···, “0–4” at time 5*t*, which take the values of age composition of the hypothetical stock. *t* can be regarded as one “unit” of transition time, so it can take the value of 1 for computational convenience.

The next step is to try out a series of *S* between 0 and 1 and find out which value can best pull the actual and hypothetical stock curves together. A high *S* close to 1 implies a high retention rate in the “mobile-to-settled” transition, with early flow cohorts gradually getting old and their child and elderly dependents joining them. In contrast, a small *S* close to 0 suggests a low retention rate in the settlement process, with pioneer migrants leaving the destination within a short time and their dependents being unable to join them at the destinations. Admittedly, *S* is only an indicator under highly simplified assumptions. That said, it can be used to standardize and capture the overall, average situation, allowing us to numerically compare migration regimes across different countries.

## Data analysis: the “China difference” based on a cross-national comparison

The analytical framework set up above allows us to break down our two research questions on family togetherness and long-term residence into four expectations, which can be checked by comparing age profiles of migrant flow and stock of China and other countries:

1. Children and the elderly are expected to be underrepresented among migrants in China, so the child and elderly proportions of China’s flow should be low. The age profile of China’s flow should have only one peak at young adult age, whereas the others should have two peaks at young adult and young child age.
2. It is expected that Chinese migrants tend to leave the destinations when growing old, and that their old parents are discouraged from migrating to join them, so the elderly proportion of China’s stock should be low. The age profile of China’s stock should stand out with a clear peak at young adult age, in contrast to the other countries’ that are relatively flat and smooth across all ages.
3. The strong age selectivity of China’s flow is expected to remain through the transition into its stock, resulting in similar age profiles and median ages of China’s flow and stock.
4. Migrants in China are expected to experience difficulty making the “mobile-to-settled” transition; therefore, their “settlement rate” should be low.

The reference points to be used to compare with China are India, Japan, and the US. Since the age profiles of internal migrants are affected by the general age profiles of the total population in the selected countries, we briefly introduce them here (see Columns 1–4, Table 1). Among the four countries, Japan has the lowest child proportion and the highest elderly proportion of its total population, as the country has been experiencing severe population aging (Muramatsu and Akiyama 2011). In contrast, India has the highest child proportion and the lowest elderly proportion among the four. Much like China, India has massive temporary migration (Coffey 2013). Migrants in India do not face the same legal and institutional barriers to settlement as their counterparts in China. Although mobility is “free” in India, migrants’ food security, education, housing and health care needs are not well protected. Hence, though not legally differentiated and circumscribed as in China, internal migrants in India face other barriers and are often viewed as “outsiders” in the destinations (Rajan and Sumeetha 2020), a situation somewhat similar to that of migrants in China. Lacking formal residency rights, migrant children in India, especially those whose parents migrate seasonally, also have difficulties getting an education. The child and elderly proportions of China and the US lie in the middle of the four countries. In this section, we first explain the data sources of migrant flow and stock of the selected countries, and then examine the four expectations with reference to the data.

### Data sources

**Figure 4.**
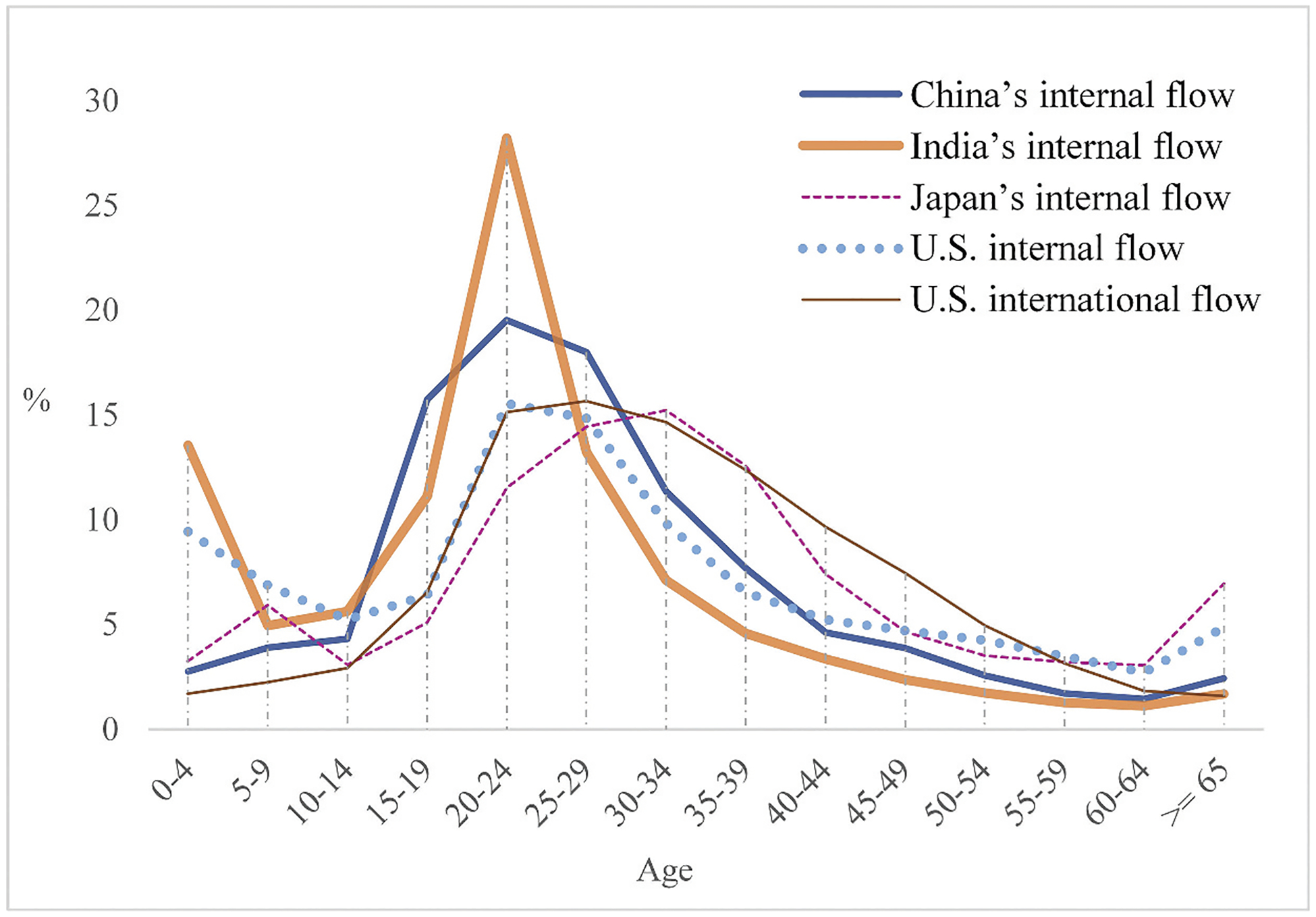
Age profiles of migrant flow of selected countries. Source: See Table 2

Cross-national analysis of migrant flow and stock is never straightforward, as the data availability and compatibility vary across different countries. We have to operate under the constraints of public available census and survey data, making needed adjustments to allow some broad comparisons so that one can see the big picture. Despite all the difficulties in gathering and deciphering migration age data especially in China (Chan and Yang 2020), we have decided to use the 2010-round data, except that China’s internal flow data are from 2000 (Table 2). 2010 is the most recent year when age data are available for all the selected countries. Also, we consider 2010 a “normal” year from which we can sketch a “representative” picture of migration and make some general arguments. That year had no special global events, such as the outbreak of Covid-19, which could otherwise severely distort migration patterns and therefore, the age profiles. No flow age data are provided in China’s 2010 census, and 2000 is the closest year for which China has that data. Admittedly, with the emergence of the “second-generation” migrant workers in recent decades in China (Chan 2010), some differences likely exist between the age patterns of 2000 and 2010. That said, as noted in the literature (see, e.g. Figure 2–4 in Duan, Yang and Ma (2012)), migrants’ age pattern in 2000 is actually quite typical of the situation in that period. As for the geographic boundaries to cross when measuring migration, we use the smallest administrative units for which migration age data are available, as suggested by Bernard, Bell, and Charles-Edwards (2014b).

In identifying the coverage of the selected countries’ flow and stock data, we start from internal migration. As stated before, the non-*hukou* migrants in China are a stock concept (Series F, Table 2). The internal flow of China (Series A, Table 2), on the other hand, includes people whose residence is different from five years earlier, regardless of their *hukou* status. That is, the flow includes both *hukou* and non-*hukou* migrants. Therefore, when estimating migrants’ “settlement rate”, we will have to make some adjustments to account for that difference in China’s flow and stock data (more details in the Data Analysis section). The census of India records the residence duration of internal migrants in the destinations, which varies from less than one year to ten years and above. We consider migrants of one to four years’ stay to be India’s flow (Series B, Table 2) because this length is more comparable to the flows of other countries. The number of migrants of any stay duration, i.e. all migrants, is India’s stock figure (Series G, Table 2). Internal migration data of Japan and the US only cover the flow but not the stock, which is common in many countries for the reasons explained before. They use time intervals of five years (Series C, Table 2) and one year (Series D, Table 2), respectively. Regarding international immigration, the U.S. international flow is the total of permanent and temporary immigrant admissions in one year (Series E, Table 2).^1^ The U.S. international stock refers to all existing foreign-born population in the country (Series H, Table 2).^2^

### Empirical analyses

We now proceed to check if the four research expectations are supported by age data.

#### Expectation I: The flow of China should have low child and elderly proportions. Its age profile should have only one peak at young adult age

China’s flow has the lowest child proportion among all internal flows of the selected countries (Column 5, Table 1). It also has the second lowest elderly proportion, only marginally higher than India’s (Column 7, Table 1). Despite the somewhat similar marginalization of Chinese and Indian migrants described above, India’s migration has a far larger proportion of children, suggesting that children are particularly underrepresented in China’s migration. Of course, the high child proportion of Indian migrants is also related to the same situation of its total population (Columns 1 and 2, Table 1). To provide a different case, we introduce Japan, whose child proportion of the total population is lower than that of China. Even so, Japan’s flow still has a higher child proportion than China’s! Furthermore, the child and elderly proportions of China’s internal flow lie between that of other countries’ internal flow and U.S. international flow, corroborating the proposition that China’s institutional barriers resemble immigration controls and regulations.

The age profiles of the internal flow of India, Japan and the US all present two peaks, one higher among young adults and one lower among young children (Figure 4). In contrast, the age profile of China’s internal flow has only one peak among young adults, missing the children’s peak. It should be noted that different time periods covering the migration (Table 2) would affect the number of children counted in the flow, specifically those whose age is younger than the period length. Compared with those of India and the US, the flows of China and Japan underestimate the number of children below 5. That said, there is still a hump after age 5 on the age profile of Japan, while the children’s peak is completely missing on that of China (Figure 4). This feature is shared with the age profile of U.S. international flow, which again points to the similarity in age between China’s internal and U.S. international flow.

#### Expectation II: The stock of China should have a low elderly proportion. Its age profile should have a relatively sharp peak instead of being flat

**Figure 5.**
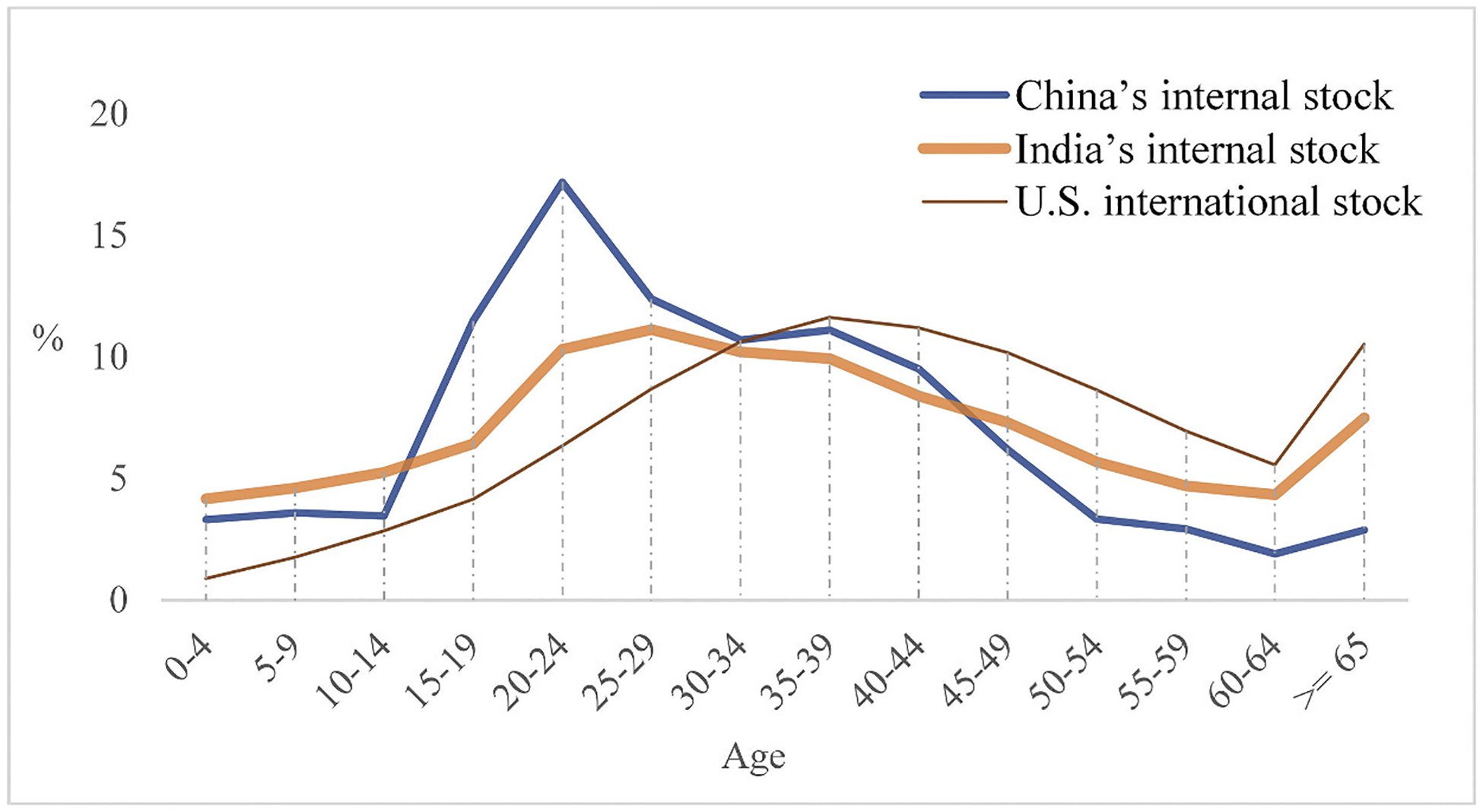
Age profiles of migrant stock of selected countries. Source: See Table 2

Compared to India’s internal and U.S. international stock, China’s internal stock has a low elderly proportion (Column 10, Table 1). India’s much higher elderly proportion of the stock is quite telling relative to its much lower proportion in the total population (Column 4, Table 1), which is thus in stark contrast to the low elderly proportion of China’s stock. Moreover, the stock age profiles of India and the US appear relatively flat, whereas that of China shows a sharp peak among young adults aged 15–39 (Figure 5).

#### Expectation III: The age profiles and median ages of China’s stock and flow should be similar

**Figure 6.**
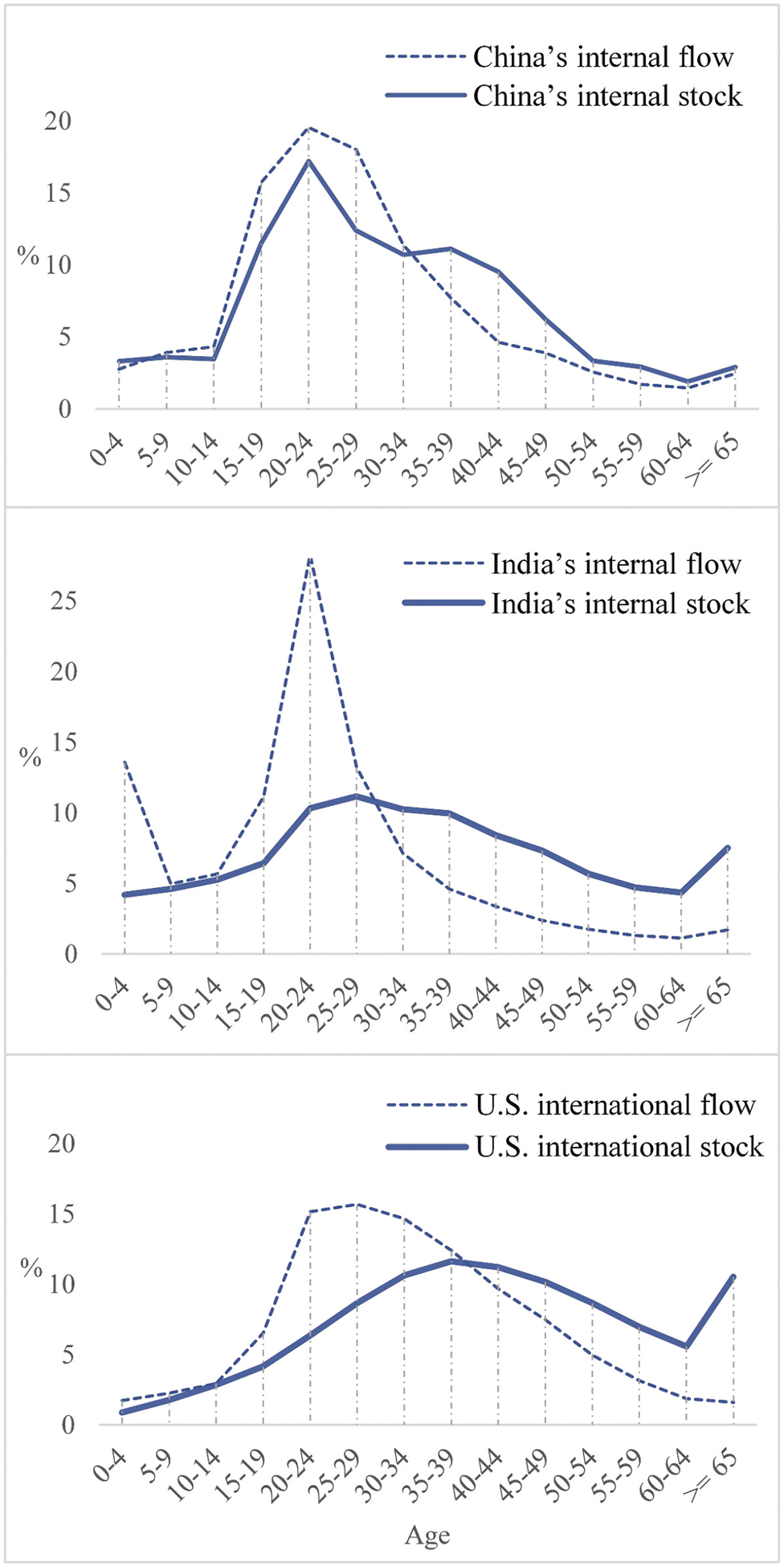
Age profiles of migrant flow and stock of selected countries. Source: See Table 2

Figure 6 shows that India’s internal migration and U.S. international immigration both have a flat and smooth stock curve as a result of the accumulation of earlier migrants and elderly dependents, despite strong age selectivity in their flows. In contrast, China’s flow and stock curves are similar, both with strong age selectivity. The above results are also consistent with the differences in the median age between migrant flow and stock of the three countries (Table 3). For China, the difference is only 2.7 years, while those of India and the US are quite large, almost 9 years!

#### Expectation IV: The “settlement rate” of migrants in China should be low

**Figure 7.**
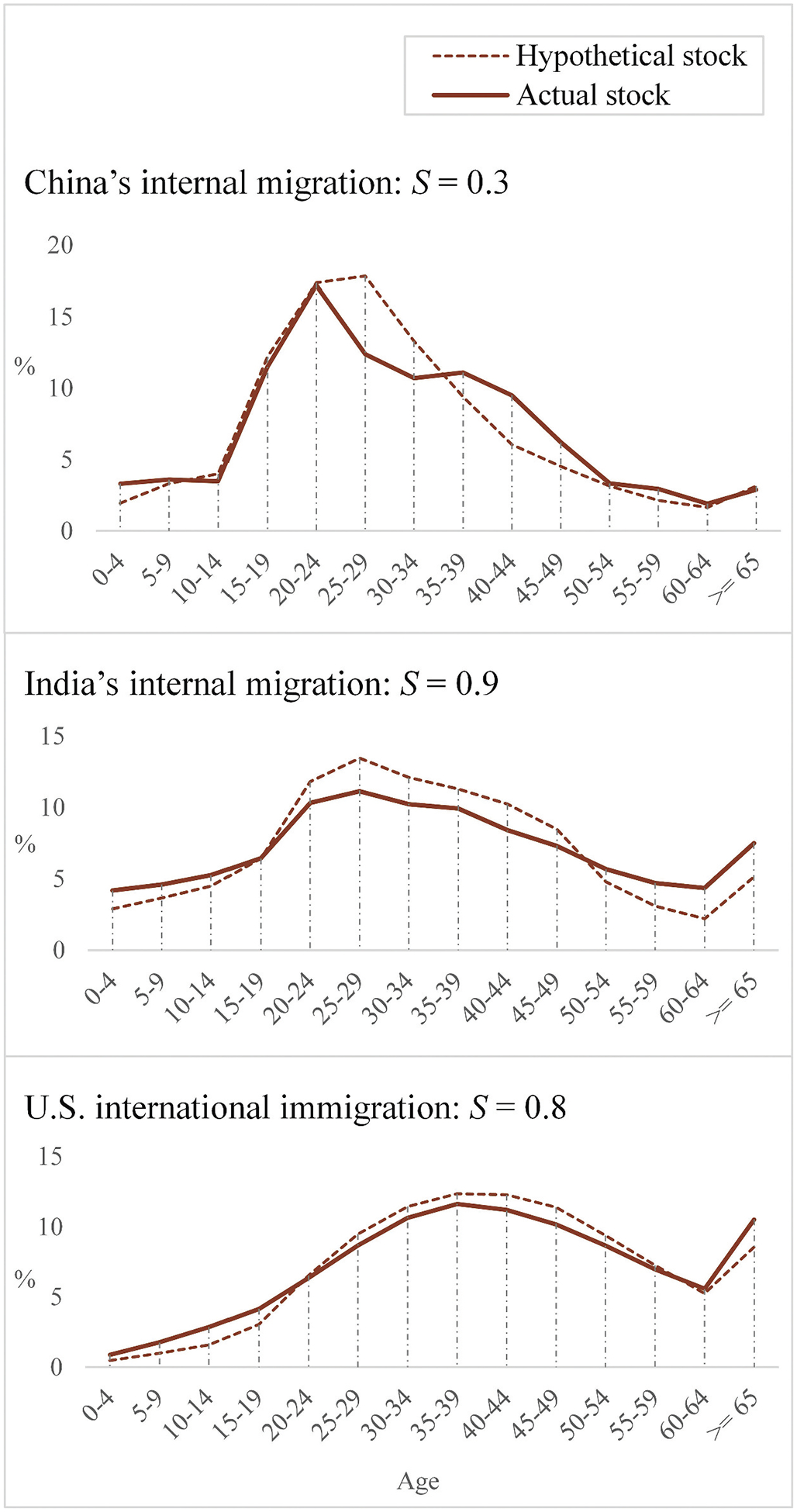
Approximation of hypothetical and actual stock curves and the resultant “settlement rate” *S*.

Based on the calculation method of the “settlement rate” explained before, the approximation of the hypothetical and actual stock curves is shown in Figure 7. The estimates of *S* for China’s and India’s internal and U.S. international migration are 0.3, 0.9, and 0.8, respectively. We will make some adjustments to *S* to account for inconsistencies in the data.

As noted earlier, China’s stock covers only non-*hukou* migrants, while its flow covers both *hukou* and non-*hukou* migrants. We adjust the “settlement rate” of China to exclude *hukou* migrants. Since the national *hukou* conversion rate is not published, we have decided to only look at the *hukou* conversion rate of rural migrants in urban areas, as the data are available and the main direction of migration in China is rural-urban (Ma, Duan, and Guo 2014). Chan (2012) tallies that the rural-to-urban net migration in the decade between 2000 and 2010 was approximately 90 million, in which about 30 million migrants converted their rural *hukou* to urban *hukou*. Because urban-to-rural migration was minimal (Ma, Duan, and Guo 2014), the 90 million can be considered rural-to-urban migrants only. In doing so, the *hukou* conversion rate is about 33.3% for rural migrants during 2000–2010. With that, we can make a correction to *S* to exclude *hukou* migration:

(6) $$S^{\prime }=\frac{S}{1-h}$$

where *S*’ is the adjusted “settlement rate”, and *h* is the *hukou* conversion rate. The result of *S*’ is 0.45, increased from 0.3 of China’s *S*.

Adjusted for this inconsistency, we proceed to account for different time coverage of the selected countries’ flows. This type of adjustment can be quite complicated according to Rogers, Raymer and Newbold (2003). Given the main purpose of this study, we omit the complexities and derive a crude proxy using our rationale of calculating the “settlement rate”. We suppose that different time periods over which migration is measured can be approximated to different extent of migrant “attrition” resulted from “unsettlement”, with longer periods corresponding to greater “attrition”. This allows us to align different time periods based on the estimating process of *S* specified in equations (1)–(5). What is different here is that instead of using age-specific *l*, we suppose *l* is the same for all age groups.

The time periods covering China’s and India’s internal migration and U.S. international immigration are 5 years, 1 to 4 years, and 1 year, respectively. We will align the latter two with that of China. When aligning the U.S. case, from a 1-year *S* to a 5-year *S*, there is a difference of 4 years. We borrow and adjust equation (5) to reflect this time difference:

(7) K4t=l+lSt+lS2t+lS3t+lS4t

which can be turned into:

(8) K4t=l(1+St+S2t+S3t+S4t)

We plug in *t*= 1 for the equation, because in this way _4*t*_*K* indicates the aggregated population after 4 years’ attrition of migrants. We also plug in *l*= 1, so that _4*t*_*K* can be viewed as an accumulated settlement “chance”, which is a ratio rather than a specific number of settled population. We then plug in the previously estimated U.S. *S* = 0.8 to compute a _4*t*_*K*. Finally, the accumulated “chance” _4*t*_*K* is standardized to a “rate” through being divided by 5. In short, by estimating the attrition of migrants over 4 years, a 1-year *S* is transferred to a 5-year *S*. The adjusted *S* of the US is 0.67.

To adjust the Indian case, we first do a reverse procedure of the above to transfer the 3-year (1–4 years’ time period suggests a moving window of 3 years) *S* to a 1-year *S*, and then follow the same procedure set above to transfer the 1-year *S* to a 5-year *S*. The adjusted *S* of India is 0.81.

From our results, the *S* of China’s internal migration (0.45) is much lower than that of India’s internal (0.81) and U.S. international migration (0.67). These results show that the proportion of China’s internal migrants who settle in the destinations after a long time since their initial migration is only about 45%, while for India’s internal and U.S. international migrants, they are 81% and 67%, respectively. The commonality between China’s internal and U.S. international flow demonstrated earlier suggests a similarly strong selectivity of migrants regarding age in these two cases. Yet further scrutiny in this part shows that although U.S. international immigration is strongly selective at the beginning (i.e. the strong selectivity of young adults of the flow), those who already migrate have a larger chance of long-term residence (i.e. the larger chance of earlier migrants to remain in the stock). Whereas for China’s internal migration, that strong age selectivity remains almost unchanged over time, resulting in the “forever youthfulness” of the migrants.

## Discussion

Our cross-national comparisons used a series of age-related indicators, including child and elderly proportions, shapes of age profiles, median ages, and the “settlement rate”, revealing the specialty of China’s migration age patterns. It has been demonstrated that the internal flow and stock of China differ significantly from the internal and international flows and stocks of other countries. Compared to India, Japan and the US, China has an internal flow with a noticeably low share of children and the elderly. This is reflected in the low child and elderly proportions of China’s flow, as well as the absence of the children’s peak on China’s flow curve. In many respects, China’s internal flow has more in common with other countries’ international flow than with internal flows, reflective of the similarity between China’s internal institutional barriers and international border controls. As for China’s internal stock, it is even more special relative to the others. The stock curve of China has a sharp peak, and it appears surprisingly similar to its own flow curve. In contrast, India’s internal and U.S. international stock curves are rather flat, fundamentally different from their flow curves. The same pattern exists in the median ages. The median ages of China’s flow and stock are much closer to each other when compared to those of other countries. Additionally, the elderly proportion of China’s stock is quite low, the same as its flow.

These findings show that Chinese young adults are most able to migrate, while their child and elderly dependents have limited capacity to do so. Furthermore, the pronounced selection of young adults in China’s flow does not diminish as the “mobile-to-settled” transition occurs. Over time, earlier migrants who gradually grow old return to their origins rather than stay in the destinations and reunion with their families. In contrast, in other countries, the domination of young adults is not that much salient. As the years pass, those who initially migrated to the destinations grow old and are able to bring over their family members. In particular, children are much more likely to be brought by their migrant parents to the destinations in other countries. In this regard, Japan stands in marked contrast to China. The total population of Japan features a low child proportion, while its migrants have a high child proportion. Compared to that of Japan, the child proportion of China’s total population is high, whereas the child proportion among Chinese migrants is significantly lower. Lacking access to education, Chinese children tend to be left behind and attend school in the origins rather than migrate with their parents to the destinations. Indian children also have problems accessing education in the destinations, but they are more likely to accompany their parents and migrate. Instead of attending school, many of them care for younger siblings or become child laborers in the destinations (Rajan and Sumeetha 2020). The above findings are numerically verified by the “settlement rate” *S*. In comparison to migrants in other countries, the percentage of those who make the “mobile-to-settled” transition in China is much lower. It turns out that the *S* of China’s internal migrants is not only much smaller than that of internal migrants in India, but also smaller than that of international immigrants in the US. This suggests that Chinese internal migrants’ overall chance of long-term residence in the destinations is fairly low, consistent with the narrative that China’s internal migration is largely circular. It is worth noting that in the context of international circular migration, the halt of labor migration schemes and the surge of border enforcement can lead to a decrease in circular migration and an increase in migrant settlement. For example, following the cessation of the Turkey-to-Germany guestworker program in 1973, there was an increase in the number of Turkish immigrants who stayed for extended periods and reunited with their families (Hess and Green 2016). Likewise, the termination of a long-standing guestworker program in 1964 and the subsequent escalation of border militarization in the US resulted in reduced return migration and increased settled families of Mexicans (Massey and Pren 2012). These cases show the efficacy of China’s institutions in preventing settlement. Despite border enforcement’s ability to restrict entry, it does not deter and in fact facilitates settlement.

With the above analyses, our two research questions are answered. That is, it is difficult for migrants in China to achieve family togetherness and long-term residence. Thus, many of them migrate temporarily instead of settling permanently. It is apt to use Swider’s (2011) “permanent temporariness”, based on her study of construction workers in China, to characterize the general situation of migrants in China, as they are separated from family in their hometown and excluded from the destination society simultaneously.

## Conclusion

There is nothing new that migration is age-selective, but the age patterns of China’s internal migration tell us not only the demographics of migrants but also the underlying mechanisms of its migrant labor regime, a vital contributor to China’s economic success in the last four decades. Using other countries’ internal and international migration age profiles as reference points, this paper disentangles the distinctive feature of China’s internal migration – the “forever youthfulness” of migrants. The major destinations, typically the coastal industrial and trade hubs where migrants flock to, keep “recycling” young working-age adults. With new young workers continuously replacing older cohorts, the destinations’ young labor supply is almost indefinite. These cities make use of the abundance of young migrant labor, producing cheap commodities that are super-competitive in global markets. Barred by institutions especially the *hukou* system, migrants in these cities face tremendous difficulties settling there. For most migrants – specifically, the non-*hukou* migrants – migration is permanently temporary and does not lead to settlement. While this is a “low-cost” development strategy that clearly saves costs of social reproduction for the coastal cities and provinces where migrant workers concentrate, the costs are offloaded onto migrant-sending provinces and migrant families themselves. In fact, there is a rather unequal exchange among provinces set up by such a migration regime, which has reinforced the underdevelopment of many inland, less-urbanized provinces (The Economist 2015; Yang and Chan 2023). This is a topic deserving research in understanding China’s geography of development.

Methodologically, to examine the link between migration and settlement through the lens of age, this paper has developed a series of age-related indices. We drew on the concepts of migrant “flow” and “stock”, which had never been connected to migrant settlement previously, and derived a framework to measure the “mobile-to-settled” transition numerically. Using age data of migrant flow and stock of several countries, we showed that the inability of China’s internal migrants to settle usually comes from two sources, the difficulties in maintaining migrant family togetherness, and the impediments to long-term residence of migrants themselves. Regarding the former source, China’s internal migration is overwhelmingly dominated by working-age adults, while children and the elderly are underrepresented in it. As for the latter, as earlier migrants grow older, they are more likely to return to the origins than to stay in the destinations. These findings are consistent with what other researchers have reported on China’s internal migration with different data. For example, Chan and Ren (2018) drew on China’s 2015 mini-census and found that while there is a small percentage of children of migrants in the destinations, the great majority of them are “left-behind” in the origins. Moreover, Cheng and Zhu (2021) used survey data from the National Health Commission of China and argued that as migrants grow older, significantly more of them go back to the origins. All these point to the family-unfriendly environment for migrant families in China.

Obviously, limited data availability has prevented us from conducting a full-fledged, systematic cross-national comparison to attain better numerical estimates. That said, arguably, the framework and indicators developed in the paper show the heuristic value of our broad approach based on age data. Age data have the advantage of wider coverage and easy accessibility compared to costly sample surveys often used to measure migrant settlement. Moreover, the methods we developed can be extended to examine migrant settlement outcomes more generally and in different situations, with relevance to both internal and international migration. In particular, the “settlement rate” is an indicator of the proportion of migrants who ultimately stay in the destinations in the long term. It can be used to gauge the success or failure of a program to integrate migrants in a certain destination, a policy concern to many local governments interested in integrating newcomers. We believe that the various concepts and methods proposed in the paper open a new avenue for future research in harnessing age data to study the spatial politics of migrant labor, family migration, structural inequalities migrants face, and many other related topics.

## Figures and Tables

**Table 1.** Percentages by age group of total population, migrant flow and stock.

	Total population						Migrant flow				Migrant stock		
Country	Children (age 0–14)	Children (age 5–14)	Young adults (age 15–39)	The elderly (age 65+)	Migration type	Country	Children (age 5–14)^a^	Young adults (age 15–39)	The elderly (age 65+)	Country	Children (age 0–14)	Young adults (age 15–39)	The elderly (age 65+)
China	16.61	10.94	40.78	8.92	Internal	China	8.21	72.34	2.44	China	10.37	62.87	2.89
India	30.87	21.52	42.04	5.49		India	10.59	64.27	1.70	India	14.04	48.03	7.50
Japan	13.22	9.05	29.83	23.01		Japan	9.01	58.90	6.95				
US	20.41	13.37	33.92	12.69		US	12.11	53.16	4.86				
					International	US	5.19	64.41	1.59	US	5.50	41.43	10.51
*Column*	*1*	*2*	*3*	*4*			5	6	7		*8*	9	*10*

Source: See Table 2

a Different time periods covering the migration would affect the proportion of children whose age is younger than the period length. To remove the effect of such inconsistency, we compute child percentage for those aged 5 or over.

**Table 2.** Data sources of migrant flow and stock of selected countries.

	Migrant flow					Migrant stock		
Migration type	Internal				International	Internal		International
Country	China^a^	India^b^	Japan^c^	US^d^	US^e^	China^f^	India^g^	US^h^
Time of survey	2000	2011	2010	2010	2010	2010	2011	2010
For flow: Time period covering the migration For stock: Duration of stay in destinations	5 years	1 to 4 years	5 years	1 year	1 year	≥ 6 months	All	All
*Series*	*A*	*B*	*C*	*D*	*E*	*F*	*G*	*H*

Source:

a National Bureau of Statistics, China (2002). *2000 Decennial Census*.

bg Office of the Registrar General & Census Commissioner, India. *2011* *Decennial Census*.

c National Statistics Center, Japan. *2010* *Quinquennial Census*.

d U.S. Census Bureau (2010). *Current Population Survey, 2010 Annual Social and Economic Supplement*.

e Office of Immigration Statistics, U.S. Department of Homeland Security. *Yearbook of Immigration Statistics: 2010*.

f National Bureau of Statistics, China (2012). *2010 Decennial Census*.

h Population Division at the Department of Economic and Social Affairs, United Nations. 2011. *Trends in International Migrant Stock: Migrants by Age and Sex*.

**Table 3.** Difference of median age of the flow and stock of selected countries.

	Median age		
	Flow (age 5+)^a^	Stock	Difference
China’s internal migration	26.6	29.4	2.7
India’s internal migration	25.0	34.0	8.9
U.S. international immigration	32.5	41.4	8.9

Source: See Table 2

a For those aged 5 and over only.

## Footnotes

1. The international flow of the US excludes asylees because their age composition is not completely disclosed. They constitute only a negligible 0.16% of the flow volume.

2. There are some inconsistencies between U.S. international flow and stock data. The flow only covers authorized immigrants, while the stock covers the foreign-born, which includes both documented and undocumented immigrants. There is no public-available data to help iron out the inconsistencies. That said, as demonstrated in this paper, the age pattern of China’s internal migration appears quite distinct. We expect that such differences do not have too much influence on the broad comparison, which is meant to highlight the uniqueness of China.
